# Decreased availability of antimalarials in the private sector following the policy change from chloroquine to sulphadoxine-pyrimethamine in the Kilombero Valley, Tanzania

**DOI:** 10.1186/1475-2875-5-109

**Published:** 2006-11-14

**Authors:** Manuel W Hetzel, June J Msechu, Catherine Goodman, Christian Lengeler, Brigit Obrist, S Patrick Kachur, Ahmed Makemba, Rose Nathan, Alexander Schulze, Hassan Mshinda

**Affiliations:** 1 Department of Public Health and Epidemiology, Swiss Tropical Institute, P.O. Box, CH-4002 Basel, Switzerland; 2 Ifakara Health Research and Development Centre, Ifakara, Tanzania; 3 Health Policy Unit, London School of Hygiene and Tropical Medicine, London, UK; 4 U. S. Public Health Service, Centers for Disease Control and Prevention, Atlanta, Georgia, USA; 5 Novartis Foundation for Sustainable Development, Basel, Switzerland

## Abstract

**Background:**

Malaria control strategies emphasize the need for prompt and effective treatment of malaria episodes. To increase treatment efficacy, Tanzania changed its first-line treatment from chloroquine to sulphadoxine-pyrimethamine (SP) in 2001. The effect of this policy change on the availability of antimalarials was studied in rural south-eastern Tanzania.

**Methods:**

In 2001 and 2004, the study area was searched for commercial outlets selling drugs and their stocks were recorded. Household information was obtained from the local Demographic Surveillance System.

**Results:**

From 2001 to 2004, the number of general shops stocking drugs increased by 15% and the number of drug stores nearly doubled. However, the proportion of general shops stocking antimalarials dropped markedly, resulting in an almost 50% decrease of antimalarial selling outlets. This led to more households being located farther from a treatment source. In 2004, five out of 25 studied villages with a total population of 13,506 (18%) had neither a health facility, nor a shop as source of malaria treatment.

**Conclusion:**

While the change to SP resulted in a higher treatment efficacy, it also led to a decreased antimalarial availability in the study area. Although there was no apparent impact on overall antimalarial use, the decline in access may have disproportionately affected the poorest and most remote groups. In view of the imminent policy change to artemisinin-based combination therapy these issues need to be addressed urgently if the benefits of this new class of antimalarials are to be extended to the whole population.

## Background

The first and foremost malaria control strategy promoted by the World Health Organization (WHO) and adopted by most African countries emphasizes the need for treatment of malaria episodes with an efficacious drug within 24 hours after onset of symptoms [1,2]. African heads of state agreed at the Abuja summit in April 2000 to ensure that by 2005 at least 60% of those suffering from malaria have access to affordable, appropriate and timely treatment [3].

However, in most areas of sub-Saharan Africa, this target is still far from being reached. Many malaria patients do not receive prompt and effective treatment for malaria, even if efficacious drugs are available on the local market [4-6] or at health facilities [7]. A fever episode, especially in a child, often prompts action and very high treatment rates (over 90%) have been reported [8]. On the other hand, household surveys in 28 African countries have shown that on average only 42% of children under five years of age with fever were treated with an antimalarial. In 80% of these cases chloroquine was used, which can not be considered any more an efficacious treatment in most areas [9]. While in case of a malaria attack many factors influence care seeking behaviour [8], one of the prerequisites for successful treatment of a malaria episode is the availability of effective antimalarial drugs close to where the episode occurs.

Self-treatment at home is often the first response to a malaria episode. In many of the studies reviewed by McCombie [10] self-treatment was frequent in response to an episode of fever or malaria (44% of the self-treatment rates in published studies were reported to be above 50%). The same review found that almost half of all malaria episodes were exclusively treated outside the formal health care sector. Hamel [11] reported from a study in Kenya that 32% of caretakers treated their children's fever exclusively at home, with an antimalarial. Data recently collected in our field site in southern Tanzania suggest that exclusive home treatment is less common, with 76% of recently feverish children attending a health facility during their illness. However, not all of those children receive an antimalarial when visiting the health facility.

Reliance on home management and self-treatment raises crucially the issue of availability of antimalarials. In this context, the private retail sector has been shown to play an important role in the provision of drugs close to people's homes [10,12]. Shops are often preferred as first treatment choice because of better accessibility, shorter waiting times, more reliable drug stocks and lower costs [13]. The role of the retail sector in improving access to prompt malaria treatment has been recognized by WHO through its home-management of malaria (HMM) strategy [14].

The national drug policy in Tanzania was strengthened in 2003 when the Tanzania Food, Drugs and Cosmetics Act established the Tanzania Food and Drugs Authority (TFDA) as executive agency [15]. Since then the TFDA has been responsible for all regulatory aspects of drugs and other medical products in the country. The private retail sector for drugs includes two types of licensed drug shops as well as general stores. Mobile drug sellers are not common in most parts of Tanzania. Fully-fledged Part I pharmacies are headed by a pharmacist and are allowed to sell all registered prescription-only (Part I) and over-the-counter (Part II) drugs. In 2003, there were 344 Part I pharmacies in Tanzania, 60% of which were located in Dar es Salaam, and the rest scattered over other major towns [16]. Part II drug stores (known as *Duka la Dawa Baridi*) need to be staffed with a medically trained vendor and are allowed to sell over-the-counter drugs such as analgesics/antipyretics. However, in practice they sell a much wider variety of drugs. 5,666 Part II drug stores had been registered in 2003 [16]. According to Goodman (2004)[17], general shops were formally not allowed to stock any drugs in 2003 but were in practice permitted to sell common OTC drugs, such as painkillers. However, the legal position of drugs in these outlets has been unclear.

Until 2001, chloroquine was the first line antimalarial and it was designated a Part II drug, available over-the-counter at Part II drug stores. In practice, chloroquine was also tolerated in general stores, where it was widely available [12]. Since the 2001 policy change to sulphadoxine-pyrimethamine (SP) as first-line treatment and amodiaquine as second-line treatment, the first line antimalarial (but not the second-line) is prescription-only. Hence, SP can only be purchased legally in Part I pharmacies, besides being available in health facilities. In many parts of the country, SP has also been tolerated in Part II drug stores but not in general shops. These inconsistencies in applying legal regulations to the use and availability of SP have resulted in some level of confusion between government departments and in the development of malaria control strategies.

The change to SP aimed at increasing the effectiveness of malaria treatment and hence to decrease the malaria burden. However, little is known so far about the impact of this policy change in terms of availability of antimalarial drugs through different providers. Treatment effectiveness at community level is a function of many interlinked factors, not just the efficacy of the first line drug. In particular treatment must be available and accessible to the target population. This analysis assessed the change in availability and accessibility of antimalarial drugs in the private sector following the change of first-line treatment in Tanzania. The surveys were carried out in a rural Tanzanian setting in the frame of two projects on (1) access to malaria treatment (ACCESS Programme) and (2) deployment of antimalarial combination therapy (Interdisciplinary Monitoring Project for Antimalarial Combination Therapy in Tanzania – IMPACT-Tz).

## Methods

### Study area

**Figure 1:**
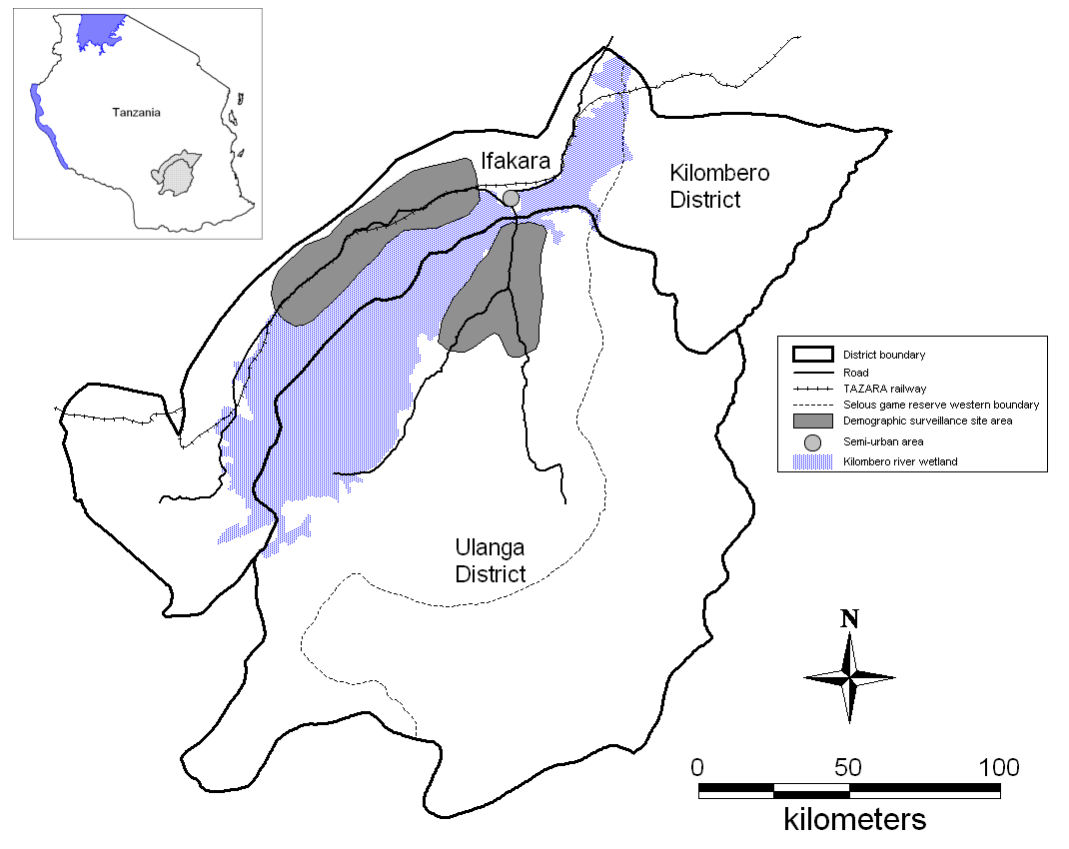
Map of Kilombero and Ulanga districts showing Ifakara town and the Demographic Surveillance System (DSS).

In 2001 and 2004, we conducted studies on antimalarial drug availability in the area of a Demographic Surveillance System (DSS) in the Kilombero and Ulanga Districts in south-eastern Tanzania (Figure 1). The DSS area covers 25 villages (13 in Kilombero and 12 in Ulanga) with almost 74,000 people (2004) in a highly malaria endemic floodplain, the Kilombero Valley [18]. Malaria transmission in the area is intense and perennial with over 300 and in some areas up to 1,000 infective bites per person per year but with seasonal fluctuations depending on rainfall patterns [19] (Killeen, personal communication). Malaria is the predominant cause of morbidity and mortality, accounting for about 50% of outpatient diagnoses at rural health facilities [18]. There were seven health facilities in the DSS area of Ulanga and another seven in the DSS area of Kilombero District in 2001 and 2004.

In 2004, the area of Ifakara town was also included in our studies. This semi-urban headquarters of the Kilombero District lies in the midst of the floodplain outside the DSS area. The population included in our studies was 45,700 in 2002 [20]. Malaria transmission is lower here, with a reported average of 29 infective bites per year [21] and a decrease in clinical incidence in recent years observed at the Designated District Hospital [22]. The study area is described in detail elsewhere [23,24].

### Shop surveys

From May to September 2001, the DSS area was searched for commercial outlets selling drugs, with the help of DSS field staff familiar with the study area and an outlet inventory from a previous study [12]. All outlets were then visited by interviewers who recorded the outlets' locations and their drug stocks. Survey methods are described in detail elsewhere [17]. Data collection was completed before the new antimalarial drug policy with SP as first line drug was implemented in the area.

A further survey was done 3 years after the policy change, in May – June 2004. The outlet list was updated after consulting village leaders and DSS field staff. All outlets were visited again by interviewers who geo-located them using a hand-held GPS unit (Garmin^® ^e-Trex^®^, Garmin Ltd.) and administered a questionnaire on drugs stocked, and other parameters not presented in this paper. In each survey, the questionnaires contained a checklist for common drug brand names obtained during pilot studies in local shops. Drugs were recorded as "in stock" only if the interviewer was shown the stock. Field supervisors checked the completed questionnaires and helped to resolve any queries. In 2004, the survey was extended to Ifakara town, where the outlets were identified with the help of local community leaders.

### Follow-up on SP availability

A follow-up study was done in November 2004 to find the reasons for the low availability of the first-line antimalarial SP. For this purpose, 50 general shops were randomly sampled from all general shops that had stocked drugs or had sold drugs previously (n = 474), weighted by the total number of shops in each of the three sampling units: Kilombero DSS, Ulanga DSS and Ifakara town. A second set of 50 shops was sampled as backup for shops temporarily or completely closed down. The shops were visited and a semi-structured questionnaire was administered to record stock of SP drugs, customer demand and wholesale sources for antimalarial drugs; if no SP was stocked, the reasons for this were elicited.

### Household and health facility information

Demographic data as well as household GPS coordinates were derived from the core DSS database. Since 1997 DSS field workers have collected basic household information (births, deaths, migrations) three times per year, i.e. every household is visited every four months. Locations of DSS households and health facilities were recorded during routine DSS data collection and ACCESS Programme activities. While precise population data were available for all years, GPS locations of households had been recorded only once in 2001 and not updated since. GPS data were available for 12,005 households (total of all households in 2004: 16,220).

### Ethical approval and informed consent

Ethical approval for the studies done within the ACCESS Programme and IMPACT-Tz was received from the institutional review board of the Ifakara Health Research and Development Centre and the Tanzanian National Medical Research Coordinating Committee (NIMR/HQ/R.8a/Vol.IX/236). In addition, IMPACT-Tz was approved by the review board of the U.S. Centers for Disease Control and Prevention. Participation was voluntary and interviewees were informed about the purpose and nature of the research. Oral informed consent was obtained prior to the interviews. Village authorities of the study area were informed about all research activities before their onset.

### Data entry and analysis

Data were double entered using Microsoft FoxPro and Microsoft Access (Microsoft Corp. Seattle, USA) and checked for coding errors and consistency. Intercooled Stata 8.0 (Stata Corp., College Station, TX, USA) was used for analysis. Mapping of shops and households was done using MapInfo Professional 7.0 (MapInfo Corporation).

## Results

### Drug stocking outlets

In 2001, 350 shops from an initial list of 439 outlets (80%) were interviewed, of which 287 stocked drugs on the day of the visit; these comprised 10 Part II drug stores and 277 general shops, but no Part I pharmacy. Of those not interviewed, 86 had closed permanently, 1 was temporarily closed, 1 refused, and for 1 no reason was recorded.

In 2004, 758 commercial outlets were visited, which were either listed in 2001 or reported by the community to have opened since then. In 625 (82%) of them, the shop-keeper was interviewed. 123 shops had closed down completely, another eight temporarily. Two shop keepers refused the interview. A total of 195 interviewed shops were in Ifakara and 430 in the DSS area. The interviewed shops ranged from little shacks with a grass-thatched roof to nicely furnished shops with brick walls and display cabinets made of glass.

In the DSS area in 2004 we recorded 19 Part II drug stores and 318 general shops stocking any type of drugs on the day of the visit (78% of interviewed shops). A further 16 outlets reported having sold drugs during the last month but were out of stock the day of the interview. As in 2001, there was no Part I pharmacy in these villages.

In Ifakara town, there were 10 Part II drug stores and 142 general shops stocking drugs in 2004. One Part I pharmacy and the hospital pharmacy of St. Francis Designated District Hospital were not included in the survey. An additional 7 general shops had sold drugs during the last month but were out of stock on the day of the interview.

Between 2001 and 2004, the total number of drug-selling shops increased in the DSS area. There were 15% more general shops stocking drugs (277 vs. 318) and the number of drug stores nearly doubled, from 10 to 19. In both years the absolute number of shops was higher in Kilombero than in Ulanga District, and the increase varied also considerably between the two districts: The number of general shops stocking drugs increased 4% in Ulanga and 23% in Kilombero. The number of drug shops doubled in Kilombero but remained almost unchanged and low in Ulanga (Table 1). An analysis of these data in relation to population numbers is shown below.

**Table 1:** Number of shops selling drugs and antimalarials.

**Location**	**Population**		**General shops stocking drugs**			**Drug Stores**			**General shops stocking AM**			**Total of AM stocking shops**		
	2001	2004	2001	2004	Change (%)	2001	2004	Change (%)	2001	2004	Change (%)	2001	2004	Change (%)

**Ulanga DSS**														
Idunda	1736	1837	6	5	-17	0	0		1	0	-100	1	0	-100
Igota	1419	1533	6	6	0	0	0		2	0	-100	2	0	-100
Igumbiro	2056	2311	9	10	11	0	0		1	2	100	1	2	100
Iragua	3547	3704	6	8	33	0	1		1	1	0	1	2	100
Kichangani	3119	3103	13	13	0	0	0		1	0	-100	1	0	-100
Kidugalo	1695	2539	7	3	-57	0	0		4	0	-100	4	0	-100
Kivukoni	5634	5612	22	25	14	1	0	-100	4	3	-25	5	3	-40
Lupiro	3591	4009	24	23	-4	1	1	0	0	2		1	3	200
Mavimba	2268	2417	11	15	36	0	0		1	1	0	1	1	0
Milola	1277	1282	3	5	67	0	1		1	0	-100	1	1	0
Minepa	1955	1964	9	7	-22	0	0		2	1	-50	2	1	-50
Nakafulu	1079	919	6	7	17	0	0		0	0		0	0	
**Sub-total**	**29376**	**31230**	**122**	**127**	**4**	**2**	**3**	**50**	**18**	**10**	**-44**	**20**	**13**	**-35**
**Kilombero DSS**														
Idete	4657	4661	9	18	100	1	1	0	1	0	-100	2	1	-50
Igima	3210	3793	17	21	24	1	4	300	12	3	-75	13	7	-46
Ikule	1571	2244	10	12	20	1	3	200	4	0	-100	5	3	-40
Kisegese	1113	1370	5	9	80	0	0		3	2	-33	3	2	-33
Lukolongo	3526	3821	9	6	-33	0	0		1	1	0	1	1	0
Mbingu	4928	5380	14	24	71	1	1	0	7	5	-29	8	6	-25
Mchombe	4006	4452	30	37	23	2	3	50	9	0	-100	11	3	-73
Miwangani	1446	1702	7	9	29	0	0		2	2	0	2	2	0
Mkangawalo	4426	4675	15	16	7	1	1	0	9	0	-100	10	1	-90
Mngeta	3218	3399	14	13	-7	0	2		2	1	-50	2	3	50
Mpofu	1673	1897	1	1	0	0	0		0	0		0	0	
Namawala	2799	3675	19	19	0	1	1	0	5	0	-100	6	1	-83
Njagi	1597	1678	5	6	20	0	0		2	0	-100	2	0	-100
**Sub-total**	**38170**	**42747**	**155**	**191**	**23**	**8**	**16**	**100**	**57**	**14**	**-75**	**65**	**30**	**-54**
**Total DSS**	**67546**	**73977**	**277**	**318**	**15**	**10**	**19**	**90**	**75**	**24**	**-68**	**85**	**43**	**-49**
Ifakara*		**45726**		**142**			**10**			**5**			**15**	

Data for the DSS villages of Kilombero and Ulanga Districts and Ifakara town. Ifakara population: National census 2002.

### Availability of antipyretics and antimalarials in general shops

In 2001 and 2004, 99% of general shops stocking drugs in the DSS had antipyretics/analgesics in stock. In 2004, the main products stocked in these outlets were paracetamol generics (93%), aspirin (79%) or co-formulations of paracetamol and another antipyretic/analgesic compound (25%). One particular paracetamol generic made in Tanzania (Sheladol™, Shelys Pharmaceuticals Ltd.) was found in 205 (64%) general shops.

In 2001, of the 277 general shops stocking drugs, 75 (27%) had an antimalarial in stock. All of these stocked chloroquine, while only 1% of all general shops stocked SP and less than 1% amodiaquine or quinine. None stocked injectable antimalarials.

In 2004, of the 318 general shops stocking drugs only 24 (8%) had an antimalarial in stock. 5% stocked amodiaquine, 3% SP and 1% quinine. One general shop had injectable quinine in stock.

In Ifakara in 2004, all except one of the 142 general shops stocking drugs had antipyretics/analgesics in stock. Paracetamol generics (89%) and co-formulations of paracetamol and another antipyretic/analgesic compound (49%) were more frequently stocked than aspirin (13%). Only five (4%) general shops stocking drugs had an antimalarial in stock, all of them amodiaquine.

Hence, within 3 years of the policy change from chloroquine to SP, the number of general shops that stocked antimalarials decreased by 68%, despite an increase in the number of general shops stocking drugs. The decrease was more marked in Kilombero (75%) than in Ulanga DSS area (44%). The proportion of shops stocking drugs that had antipyretics/analgesics remained unchanged. This resulted in a considerable increase in the number of general shops offering treatment for fever – but no cure for malaria.

### Availability of antipyretics and antimalarials in drug stores

All Part II drug stores in the DSS area stocked antipyretics/analgesics as well as antimalarial drugs in 2001 and 2004. In 2004, they all stocked paracetamol generics and 84% stocked aspirin and diclofenac, a non-steroidal anti-inflammatory drug.

In 2001, the antimalarials stocked by the 10 drug stores in the DSS area were chloroquine (80%), SP (70%), quinine (70%) and amodiaquine (60%). 80% stocked also an injectable antimalarial.

In 2004, the 19 drug stores stocked mainly amodiaquine (95%), SP (90%), and quinine (74%). 68% stocked an injectable antimalarial, usually quinine.

In Ifakara in 2004, all of the 10 drug stores stocked paracetamol, 70% diclofenac generics and 40% aspirin. All drug stores had SP and amodiaquine and 80% had quinine in stock. 30% stocked an injectable antimalarial. Other antimalarial drugs, such as mefloquine or artesunate were only rarely found (Table 2).

**Table 2:** Products stocked by drug stores and general shops in the study area in 2004

**(a) Drug stores**	**Number of drug stores with product in stock (percentage)**															
**Location**	**SP**		**AQ**		**QU**		**ART**		**Other**		**INJ**		**Any AM**		**Any AP**	

Ulanga DSS (*N *= 3)	3	(100)	3	(100)	3	(100)	0	(0)	0	(0)	3	(100)	3	(100)	3	(100)
Kilombero DSS (*N *= 16)	14	(88)	15	(94)	11	(69)	0	(0)	1*	(6)	10	(63)	16	(100)	16	(100)
Total DSS villages (*N *= 19)	17	(89)	18	(95)	14	(74)	0	(0)	1	(5)	13	(68)	19	(100)	19	(100)
Ifakara (*N *= 10)	10	(100)	10	(100)	8	(80)	2	(20)	1**	(10)	3	(30)	10	(100)	10	(100)
**Total **(*N *= 29)	**27**	**(93)**	**28**	**(97)**	**22**	**(76)**	**2**	**(7)**	**2**	**(7)**	**16**	**(55)**	**29**	**(100)**	**29**	(100)

**(b) General shops**	**Number of general shops with product in stock (percentage)**															

**Location**	**SP**		**AQ**		**QU**		**ART**		**Other**		**INJ**		**Any AM**		**Any AP**	

Ulanga DSS (*N *= 127)	5	(4)	5	(4)	0	(0)	0	(0)	0	(0)	0	(0)	10	(8)	125	(98)
Kilombero DSS (*N *= 191)	5	(3)	10	(5)	3	(2)	0	(0)	0	(0)	1	(1)	14	(7)	188	(98)
Total DSS villages (*N *= 318)	10	(3)	15	(5)	3	(1)	0	(0)	0	(0)	1	(0)	24	(8)	313	(98)
Ifakara (*N *= 142)	0	(0)	5	(4)	0	(0)	0	(0)	0	(0)	0	(0)	5	(4)	141	(99)
**Total **(*N *= 460)	**10**	**(2)**	**20**	**(4)**	**3**	**(1)**	**0**	**(0)**	**0**	**(0)**	**1**	**(0)**	**29**	**(6)**	**454**	**(99)**

SP = Sulphadoxine-pyrimethamine or sulphalene-pyrimethamine; AQ = Amodiaquine; QU = Quinine; ART = Artesunate; CQ = Chloroquine; INJ = Antimalarialinjection; AM = Antimalarial; AP = Antipyretics/analgesics. *Chloroquine; **Includes mefloquine and dihydroartemisinin tablets

In 2001, the then first-line drug chloroquine was the most frequently stocked antimalarial in both drug stores and general shops in the DSS area. In 2004, however, the second-line drug amodiaquine was more readily available than the first-line drug SP. The percentage of drug stores with the first-line drug in stock was nevertheless higher in 2004 (90% SP) than in 2001 (80% Chloroquine). In 2004, antimalarial injections were less frequently stocked than in 2001.

### Overall access to antimalarials

**Figure 2:**
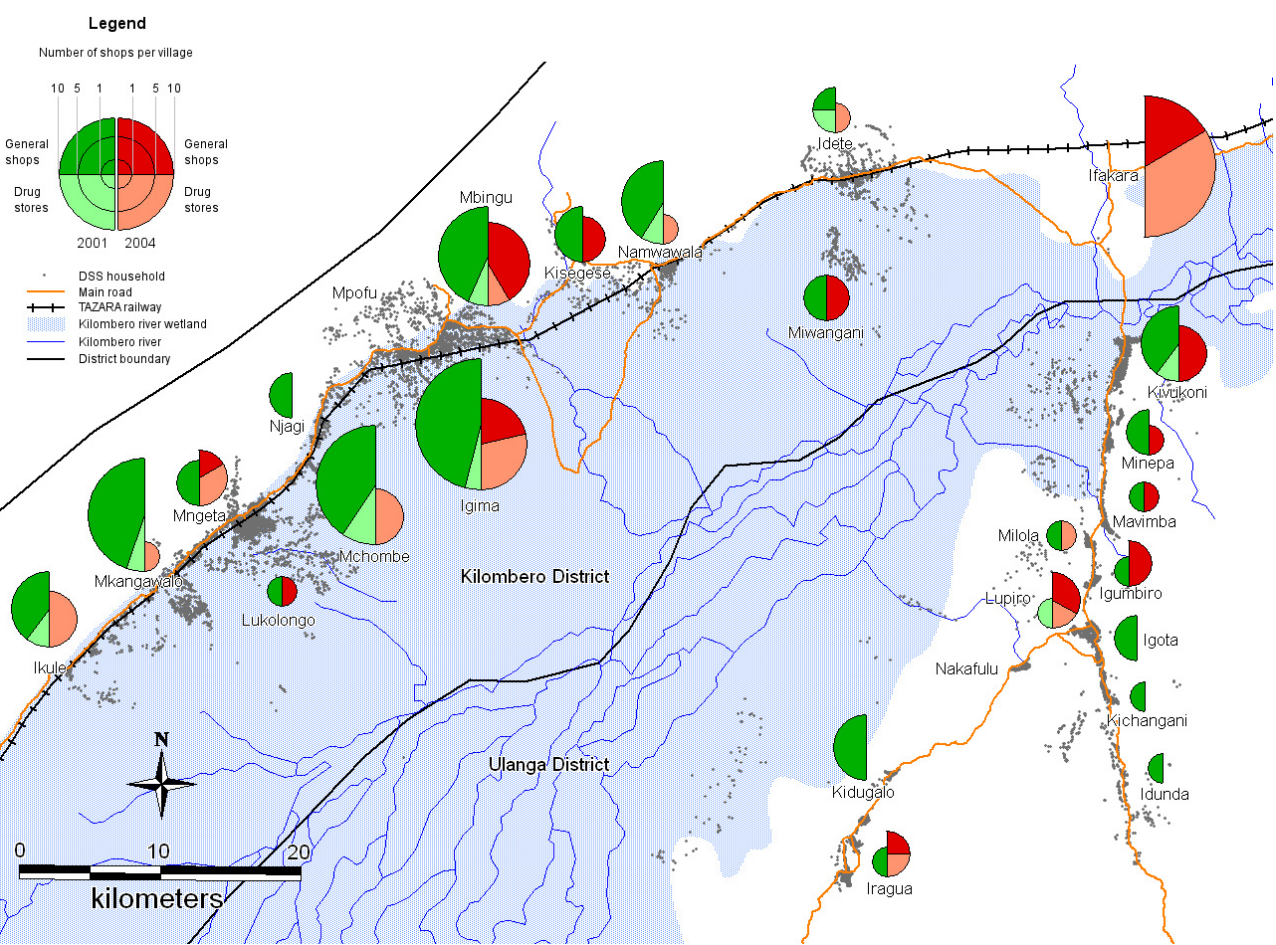
Study area with households (small dots) and number of shops stocking antimalarials, per village, in 2001 and 2004. Data for Ifakara town only available for 2004.

From 2001 to 2004, the total number of shops with antimalarials in stock (including drug stores and general shops) decreased in the DSS area by almost 50%, from 85 to 43. In 2004, more drug shops than general shops stocked antimalarials in the Kilombero DSS area and in Ifakara. By contrast, in the Ulanga DSS area, general shops stocking antimalarials outnumbered the few drug shops. The geographical distribution of antimalarial selling shops in 2001 and 2004 is displayed in Figure 2.

### Reasons for not stocking SP

Of the 50 general shops stocking drugs that were re-visited in the follow-up study, one had SP and amodiaquine, and one only amodiaquine in stock the day of the re-visit. Shops without SP stated no permission to sell (65%), lack of demand (39%), or inability to buy the drugs (23%) as reasons for not stocking SP. In total 92% of the shop-keepers were aware that they were not allowed to sell SP and 31% of shops without SP had actually tried to buy SP but could not, usually because they did not have an appropriate drug store license. These findings suggest that the respective legislation was implemented quite effectively.

Only after specific probing did 5 shop keepers (10%) mention the bad reputation or perceived side effects of SP as reasons for not stocking it. Given that shop-keepers did report customer demand for antimalarials (51% for SP, 31% for amodiaquine, 24% for quinine and 16% for chloroquine) it is obvious that there are other determinants for stocking antimalarials than only customer demand.

### Location of antimalarial selling points

While small general shops were found everywhere in the study area, shops stocking drugs were clustered in larger centres along with other resources such as permanent markets and health facilities. Linear regression analysis of the DSS data showed a significant correlation between the population of the villages and the number of shops stocking drugs (2001: R^2 ^= 0.46, *P *< 0.001; 2004: R^2 ^= 0.55, *P *< 0.001) as well as shops stocking antimalarials (2001: R^2 ^= 0.21, *P *= 0.02; 2004: R^2 ^= 0.28, *P *= 0.006). Whether or not there was a drug store in a village was significantly correlated with the size of the village in 2001 (likelihood ratio χ^2 ^= 13.14, *P *< 0.001) and 2004 (likelihood ratio χ^2 ^= 7.62, *P *= 0.006). In both years, there was no such correlation for general shops stocking antimalarials, even though the number of general shops varied with the size of the village.

**Figure 3:**
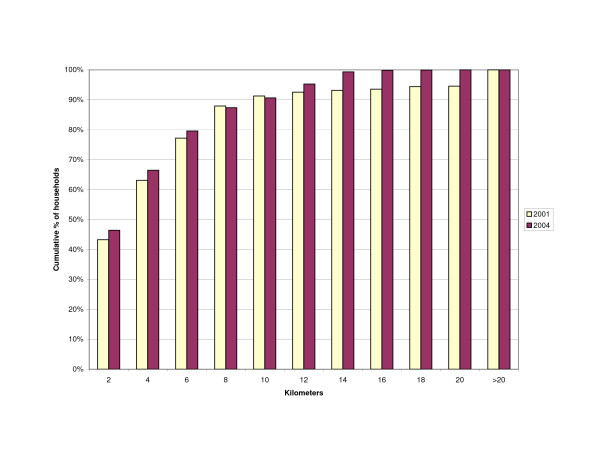
Cumulative percentage of households within given distance to nearest drug store.

**Figure 4:**
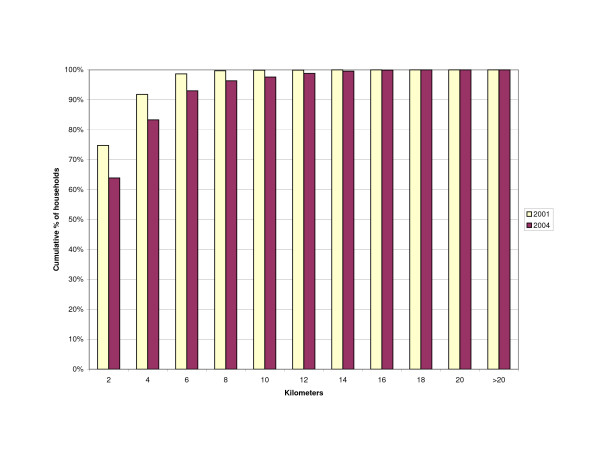
Cumulative percentage of households within given distance to nearest shop stocking antimalarials (general shops and drug stores).

**Figure 5:**
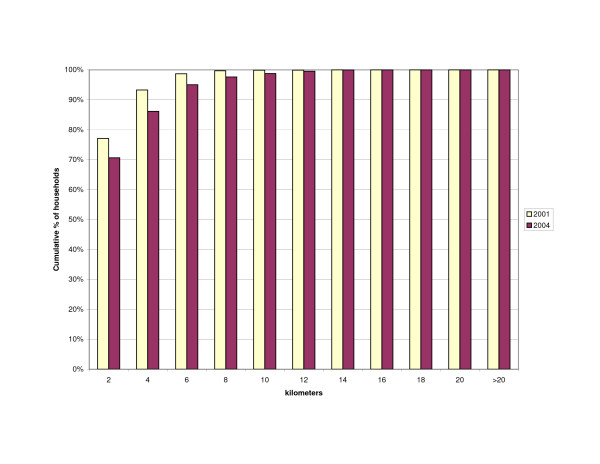
Cumulative percentage of households within given distance to any source of antimalarials (general shops/drug stores/health facilities).

The distance of households to outlets stocking antimalarials was influenced by the change in the number of outlets from 2001 to 2004. The increase in number of drug stores resulted in slightly more households living within a 2 km range of a drug store (43 vs. 46%)(Figure 3). However, the number of households within 2 km of any shop selling antimalarials decreased (Figure 4) as well as the number of households living near any source of antimalarials – shops or health facilities (Figure 5). This resulted in more households being located at a farther distance from a treatment source (Table 3).

**Table 3:** Household distance to source of antimalarials

	**Number of households (percentage)**					
	**Drug Store**		**Drug store and general shop stocking AM**		**Health facility and shops stocking AM**	

**Distance (km)**	**2001**	**2004**	**2001**	**2004**	**2001**	**2004**

0–2	5191 (43)	5571 (46)	8976 (75)	7671 (64)	9254 (77)	8479 (71)
>2–10	5766 (48)	5309 (44)	3013 (25)	4044 (32)	2735 (23)	3379 (28)
>10	1048 (9)	1125 (9)	16 (0)	290 (2)	16 (0)	147 (1)

In the DSS area the average population per shop stocking drugs decreased slightly from 235 in 2001 to 220 in 2004 due to the increase in number of shops. Similarly, the population per drug store decreased from 6'755 to 3'894, although it remained very high in Ulanga DSS (10,410 in 2004). The average for Kilombero DSS and Ifakara in 2004 was under the national average of about 6'060 persons per shop selling drugs (calculated on the basis of [16,20]). The population per shop stocking antimalarials increased considerably from 795 to 1,720 in the Kilombero DSS where the numbers of general shops stocking antimalarials had dropped most markedly. Adding health facilities to this calculation on the assumption that they have antimalarial drugs in stock does not change these ratios significantly (Table 4).

**Table 4:** Population per shop.

	**Kilombero DSS**		**Ulanga DSS**		**Total DSS**		**Ifakara**
	**2001**	**2004**	**2001**	**2004**	**2001**	**2004**	**2004**

Population	38170	42747	29376	31230	67546	73977	45726
Population per shop stocking drugs (GS & DS)	234	207	237	240	235	220	301
Population per drug store	4771	2671	14688	10410	6755	3894	4573
Population per shop stocking antimalarials (GS & DS)	587	1425	1469	2402	795	1720	3048
Population per source of antimalarials, including shops and health facilities	530	1155	1088	1562	682	1298	2690

GS = General shop; DS = Drug store

In 2001, of the 25 DSS villages, 12 had a health facility and a shop stocking antimalarials, 11 villages had only a shop. In 2 villages with a total population of 2,752 (4% of DSS) there was no antimalarial selling point at all. In 2004, 10 of the DSS villages had a health facility and a shop stocking antimalarials, eight had only a shop and two only a health facility. The number of villages without either of the two increased to five, with a total population of 13,506 (18% of total DSS population).

## Discussion & conclusion

In 2001, Tanzania embarked on a malaria treatment policy change from chloroquine to SP to increase the efficacy of malaria treatment. Four years later, the number of retail outlets stocking antimalarials had dropped to almost half the number in 2001. Furthermore, these outlets did not primarily stock the first-line antimalarial (SP) but rather the second-line drug amodiaquine. On the basis of access measures such as population per antimalarial outlet, number of villages with at least one antimalarial stockist, and distance to antimalarial stockists, availability of treatment has declined in 2004 compared to 2001.

This decrease did not affect all outlets equally. The number of Part II drug stores actually increased and in these outlets the first line drug SP was more often stocked in 2004 than chloroquine in 2001. Anecdotal evidence that some Part II drug stores opened after 2003 were running on the margins of legality was not investigated in the frame of this research, although we noticed that all except one drug seller we interviewed reported to have undergone some form of medical training. Most of them were nurse assistants with one year or less of medical training. From a regulatory point of view, the restriction to drug stores must be seen as a positive development, considering the risks of distributing antimalarials through general shops [12]. From a public health point of view, this development means that the overall number of antimalarial stocking outlets fell substantially and this bears its own risk.

Changes in availability did not equally affect the two Districts. While the relative decrease was larger in the Kilombero DSS area, the Ulanga DSS area had a lower number of shops stocking antimalarials, leaving as many as five out of 12 villages (42%; 32% of the population) without a single antimalarial retailer in 2004. Smaller villages in both districts were more at risk of losing their antimalarial retailer, as they were more likely to be served only by a general shop. However, the unit "village" may not always be absolutely adequate for such calculations, as some villages are clustered and distances between villages vary (unpublished observations). A household on the edge of one village may for example have a shop relatively close in the neighbouring village.

In Ulanga DSS in 2004, general shops stocking antimalarials outnumbered the few drug stores. The opposite was the case for the Kilombero DSS area and Ifakara, which might result in better rates of appropriate treatment in these areas – under the hypothesis that drug stores provide better services than general shops. This assumption, however, does not take into account people's perception of the different types of shops and their services, and its impact on utilisation.

The high percentage of general shops stocking antipyretic drugs (74%) suggests a considerable consumer demand for drugs against pain or fever. In addition, over 50% of general shops not stocking antimalarials reported customer demand for SP drugs. This unmet demand may be related to temporary non-availability of services and drugs at health facilities and/or longer distances to the closest facility or drug store. However, a generally good availability of SP in health facilities has been reported since the policy change [25], but stock-outs occurred recently in health facilities in the both Kilombero and Ulanga Districts.

Interestingly, chloroquine was still requested by patients who may appreciate its antipyretic effect (lacking in the case of SP). However, in 2004 only one shop was found to stock chloroquine, which was officially banned after the introduction of SP. Recent data on treatment seeking collected by the ACCESS Programme does support the observation that chloroquine has been effectively banned.

Lack of availability of antimalarials, particularly SP in general shops is clearly a result of the new drug regulations. The negative perceptions of SP because of the fear of severe side-effects, as described for Tanzania by Nsimba [26], could not be found in our studies. Interestingly, shops without antimalarials reported more demand for SP than for amodiaquine, although other shops stocked more of the latter. This may reflect the prescription-free (OTC) status of amodiaquine. Amodiaquine has never been put on the list of prescription-only drugs, so it may still be available through non-pharmacy distribution channels.

Other antimalarial drugs than the ones recommended as first-, second- or third-line treatment were basically not available at retailer level. Undoubtedly, the much higher prices of these drugs limits their availability in rural areas. In contrast to Dar es Salaam [27], artemisinin-containing monotherapies are not (yet) frequently sold in our rather remote rural study area. This is encouraging in view of the introduction of artemisinin-based combination therapy (ACT), as widespread use of monotherapies could foster resistance development [28].

As with every cross-sectional study, our surveys have the limitation of capturing a certain situation of one point in the year, ignoring seasonal changes. However, data from the follow-up survey on reasons for not stocking SP, done in November 2004, suggest that the survey data are quite representative. And since the 2001 and 2004 surveys were done in the same season, the data are at least comparable over time.

As a result of decreased availability of antimalarials, the National Malarial Control Programme's goal of improving prompt access to effective treatment may be difficult to achieve, considering the importance of the private sector in providing drugs [10,11,14]. Home-treatment of malaria with a (mostly) shop bought antimalarial was shown to be done more rapidly than bringing a child to a health facility in an area with good availability of antimalarials in shops [11]. With antipyretic drugs being available far closer to people's homes than antimalarials, the initial treatment is more likely to be done with antipyretics, potentially delaying the administration of an effective drug against malaria. This is supported by community survey data from the DSS villages and Ifakara, where on the day of illness onset, 64% of recent fever cases in children were treated with an antipyretic but only 53% with an antimalarial.

However, the interpretation of our data is complicated by the fact that data from household surveys conducted in the same areas and time periods did not show a fall in antimalarial use between 2004 and 2001 (IMPACT-Tz collaboration, unpublished data, personal communication S. Patrick Kachur). There was a (non-significant) fall in the proportion of general store users obtaining an antimalarial for febrile illness (27% to 13%), but the overall probability of obtaining an antimalarial showed a (non-significant) increase (46% to 54%). This reflected mainly an improvement in antimalarial utilisation for government facility users, and the relatively small role of general stores in antimalarial provision in 2001. The relatively unimportant role of general stores was documented by data on antimalarial volumes in 2002, which showed that general stores accounted for only 7% of all antimalarials dispensed in the DSS areas [17]. Although poorer people in these areas are no more likely to use general stores [29], it is possible that poorer groups were more affected by the change in antimalarial availability, as they were more likely to live in the most remote locations. Njau et al. [29] showed that it is the better-off who get better treatment for fever episodes. However, they spend significantly more money for the treatment they obtain from the more expensive non-governmental organisation facilities and from drug stores. Changing to better quality but also more expensive treatment sources may consequently not be an option for the poorest, unless exemption mechanisms (theoretically in place in public health facilities) increase affordability of treatment and care.

The regulatory environment that was created after the introduction of SP as first-line treatment does not support well the promotion of home-based management of malaria. This problem is likely to be further exacerbated by the imminent policy change in Tanzania with the introduction of a highly efficacious artemisinin-based combination therapy (ACT). The potential advantages of supplying ACT as prescription-only drugs through skilled providers, such as prevention of fast development of resistance and limiting irrational drug-use [30], need to be carefully weighed against the disadvantages of limiting ACT distribution to few suppliers, which may not easily be accessed by a considerable part of the population.

The Accredited Drug Dispensing Outlets (ADDO) Project which is currently being piloted in a few Tanzanian districts by TFDA and Management Sciences for Health may contribute to the solution to this problem [31]. Its goal is to improve access to affordable quality drugs and services in drug retail outlets in rural or peri-urban areas. Activities include promoting and assisting in the establishment of new drug stores even in remote areas, and in training drug sellers and shop owners to dispense a limited range of prescription-only drugs. This initiative could increase the number of shops licensed to sell antimalarials and/or ACT in the frame of a quality service provision. A big question at this time is the high price of ACTs and hence the need for subsidies to make them affordable in poor rural areas.

To assure prompt and appropriate malaria treatment, availability of effective drugs close to people's homes is essential, combined with appropriate prescription practices and improved compliance on the patient's side. In case of the imminent policy change to ACT, these issues need to be taken into consideration. A pre-requisite for this is a good coordination of the efforts of the drug regulatory and the malaria control authorities. The aim must be to guarantee that the new drugs reach all those who need them in time. A precious chance would be missed if regulatory mechanisms prevented people from having prompt access to life-saving malaria treatment with ACT.

## Authors' contributions

MWH designed and coordinated the 2004 surveys, analysed the 2004 data and drafted and finalized the manuscript. JJM and AM participated in the design and coordination of the 2004 surveys. CG was responsible for the 2001 shop survey, analysed the 2001 data and contributed to the manuscript. CL and BO contributed to the 2004 study design, data analysis and to the manuscript. SPK supervised the 2001 shop survey and contributed to data analysis, interpretation and the manuscript. AS participated in the design of the 2004 surveys and contributed to the discussion on the manuscript. RN provided the DSS household data. HM provided overall supervision and contributed to the discussion on the manuscript. The final manuscript was approved by all authors.

## References

[B1] Ministry of Health (Tanzania) (2003). National Malaria Medium Term Strategic Plan 2002–2007.

[B2] World Health Organization, UNICEF (2005). World Malaria Report 2005.

[B3] Roll Back Malaria/World Health Organization (2000). The African Summit on Roll Back Malaria, Abuja, 25 April 2000.

[B4] von Seidlein L, Clarke S, Alexander N, Manneh F, Doherty T, Pinder M, Walraven G, Greenwood B (2002). Treatment uptake by individuals infected with Plasmodium falciparum in rural Gambia, West Africa. Bull World Health Organ.

[B5] Committee on the Economics of Antimalarial Drugs, Board on Global Health (2004). Maximizing the effective use of antimalarial drugs. Saving lives, buying time: economics of malaria drugs in an age of resistance.

[B6] Nsungwa-Sabiiti J, Tomson G, Pariyo G, Ogwal-Okeng J, Peterson S (2005). Community effectiveness of malaria treatment in Uganda – a long way to Abuja targets. Ann Trop Paediatr.

[B7] Zurovac D, Ndhlovu M, Rowe AK, Hamer DH, Thea DM, Snow RW (2005). Treatment of paediatric malaria during a period of drug transition to artemether-lumefantrine in Zambia: cross sectional study. BMJ.

[B8] McCombie SC (2002). Self-treatment for malaria: the evidence and methodological issues. Health Policy Plan.

[B9] World Health Organization, UNICEF (2003). The Africa Malaria Report 2003 WHO/CDS/MAL/20031093.

[B10] McCombie SC (1996). Treatment seeking for malaria: A review of recent research. Soc Sci Med.

[B11] Hamel MJ, Odhacha A, Roberts JM, Deming MS (2001). Malaria control in Bungoma District, Kenya: a survey of home treatment of children with fever, bednet use and attendance at antenatal clinics. Bull World Health Organ.

[B12] Goodman C, Kachur SP, Abdulla S, Mwageni E, Nyoni J, Schellenberg JA, Mills A, Bloland P (2004). Retail supply of malaria-related drugs in rural Tanzania: risks and opportunities. Trop Med Int Health.

[B13] Brugha R, Zwi A (1998). Improving the quality of private sector delivery of public health services: challenges and strategies. Health Policy Plan.

[B14] World Health Organization (2005). The Roll Back Malaria strategy for improving access to treatment through home management of malaria.

[B15] United Republic of Tanzania (2003). Tanzania Food, Drugs and Cosmetics Act.

[B16] Battersby A, Goodman C, Abondo C, Mandike R (2003). Improving the supply, distribution and use of antimalarial drugs by the private sector in Tanzania.

[B17] Goodman CA (2004). An Economic Analysis of the Retail Market for Fever and Malaria Treatment in Rural Tanzania PhD-thesis.

[B18] INDEPTH Network (2002). Population and Health in Developing Countries: Population, Health, and Survival at INDEPTH Sites.

[B19] Smith T, Charlwood JD, Kihonda J, Mwankusye S, Billingsley P, Meuwissen J, Lyimo E, Takken W, Teuscher T, Tanner M (1993). Absence of seasonal variation in malaria parasitaemia in an area of intense seasonal transmission. Acta Trop.

[B20] United Republic of Tanzania (2003). 2002 Population and Housing Census.

[B21] Drakeley C, Schellenberg D, Kihonda J, Sousa CA, Arez AP, Lopes D, Lines J, Mshinda H, Lengeler C, Armstrong Schellenberg J, Tanner M, Alonso P (2003). An estimation of the entomological inoculation rate for Ifakara: a semi-urban area in a region of intense malaria transmission in Tanzania. Trop Med Int Health.

[B22] Schellenberg D, Menendez C, Aponte J, Guinovart C, Mshinda H, Tanner M, Alonso P (2004). The changing epidemiology of malaria in Ifakara Town, southern Tanzania. Trop Med Int Health.

[B23] Armstrong Schellenberg JRM, Abdulla S, Minja H, Nathan R, Mukasa O, Marchant T, Mponda H, Kikumbih N, Lyimo E, Manchester T, Tanner M, Lengeler C (1999). KINET: a social marketing programme of treated nets and net treatment for malaria control in Tanzania, with evaluation of child health and long-term survival. Trans R Soc Trop Med Hyg.

[B24] Tanner M, de Savigny D, Mayombana C, Hatz C, Burnier E, Tayari S, Deichmann U, Feachem R, Jamieson C (1991). Morbidity and mortality at Kilombero, Tanzania, 1982–88. Disease and Mortality in Sub-Saharan Africa.

[B25] (2005). Monitoring Malaria Situation and Control Activities in Tanzania, 2001–2003 Health Facility and Community Survey.

[B26] Nsimba SED (2006). How sulfadoxine-pyrimethamine (SP) was perceived in some rural communities after phasing out chloroquine (CQ) as a first-line drug for uncomplicated malaria in Tanzania: lessons to learn towards moving from monotherapy to fixed combination therapy. J Ethnobiol Ethnomedicine.

[B27] Kachur SP, Black C, Abdulla S, Goodman C (2006). Putting the genie back in the bottle? Availability and presentation of oral artemisinin compounds at retail pharmacies in urban Dar-es-Salaam. Malar J.

[B28] World Health Organization (2001). Antimalarial Drug Combination Therapy: Report of a WHO Technical Consultation.

[B29] Njau JD, Goodman C, Kachur SP, Palmer N, Khatib RA, Abdulla S, Mills A, Bloland P (2006). Fever treatment and household wealth: the challenge posed for rolling out combination therapy for malaria. Trop Med Int Health.

[B30] D'Alessandro U, Talisuna A, Boelaert M (2005). Editorial: Should artemisinin-based combination treatment be used in the home-based management of malaria?. Trop Med Int Health.

[B31] Mbwasi R Using a Holistic Approach to Transform Private Sector Drug Outlets: The Tanzania Experience.

